# Applying 12 machine learning algorithms and Non-negative Matrix Factorization for robust prediction of lupus nephritis

**DOI:** 10.3389/fimmu.2024.1391218

**Published:** 2024-08-19

**Authors:** Lisha Mou, Ying Lu, Zijing Wu, Zuhui Pu, Xiaoyan Huang, Meiying Wang

**Affiliations:** ^1^ Department of Rheumatology and Immunology, Institute of Translational Medicine, Health Science Center, The First Affiliated Hospital of Shenzhen University, Shenzhen Second People’s Hospital, Shenzhen, China; ^2^ MetaLife Lab, Shenzhen Institute of Translational Medicine, Shenzhen, Guangdong, China; ^3^ Imaging Department, Institute of Translational Medicine, Health Science Center, The First Affiliated Hospital of Shenzhen University, Shenzhen Second People’s Hospital, Shenzhen, China; ^4^ Department of Nephrology, Peking University Shenzhen Hospital, Shenzhen, China

**Keywords:** systemic lupus erythematosus, lupus nephritis, scRNA-seq, immune-related genes, NMF, machine learning, prediction model, PPI

## Abstract

Lupus nephritis (LN) is a challenging condition with limited diagnostic and treatment options. In this study, we applied 12 distinct machine learning algorithms along with Non-negative Matrix Factorization (NMF) to analyze single-cell datasets from kidney biopsies, aiming to provide a comprehensive profile of LN. Through this analysis, we identified various immune cell populations and their roles in LN progression and constructed 102 machine learning-based immune-related gene (IRG) predictive models. The most effective models demonstrated high predictive accuracy, evidenced by Area Under the Curve (AUC) values, and were further validated in external cohorts. These models highlight six hub IRGs (*CD14*, *CYBB*, *IFNGR1*, *IL1B*, *MSR1*, and *PLAUR*) as key diagnostic markers for LN, showing remarkable diagnostic performance in both renal and peripheral blood cohorts, thus offering a novel approach for noninvasive LN diagnosis. Further clinical correlation analysis revealed that expressions of *IFNGR1, PLAUR*, and *CYBB* were negatively correlated with the glomerular filtration rate (GFR), while *CYBB* also positively correlated with proteinuria and serum creatinine levels, highlighting their roles in LN pathophysiology. Additionally, protein-protein interaction (PPI) analysis revealed significant networks involving hub IRGs, emphasizing the importance of the interleukin family and chemokines in LN pathogenesis. This study highlights the potential of integrating advanced genomic tools and machine learning algorithms to improve diagnosis and personalize management of complex autoimmune diseases like LN.

## 1 Introduction

Lupus nephritis (LN) is a complex autoimmune disease affecting the kidneys and represents a significant health concern due to its diverse clinical manifestations and heterogeneous nature (1). Progress in understanding the molecular mechanism of LN has been pivotal for advancing diagnostic and therapeutic strategies.

Recent studies highlight the critical role of renal-infiltrating immune cells in driving LN pathogenesis (2, 3). Distinct subpopulations of CD4+ T helper cells are strongly associated with the immune mechanisms of LN (4). The significant accumulation of monocytes and macrophages in the renal tissues of LN patients emphasizes the pronounced involvement of immune cells in this disease (5). Recent single-cell sequencing analyses have further illuminated the relevance of monocytes as beneficial subtypes in the immune response associated with LN, highlighting their potential to modulate antigen presentation and interferon secretion (6). Exploring immune-related signaling pathways in LN patients holds promise as a novel perspective for immunotherapeutic interventions.

Single-cell RNA sequencing (scRNA-seq) technology offers an advanced approach to exploring gene expression at the individual cell level, providing profound insights into cellular diversity and biological mechanisms (7, 8). This powerful technique is particularly valuable in studying autoimmune diseases such as LN, facilitating a comprehensive exploration of the intricate cellular landscape involved in immune-driven inflammation (9). ScRNA-seq helps researchers to accurately measure gene expression within individual cells, enabling the understanding of cell heterogeneity between diseased and healthy states (10).

In parallel, the application of machine learning algorithms in biomedical research has gained significant traction (11). Machine learning, and specifically its subfield of deep learning, has shown immense promise in predictive modeling, pattern recognition, and identifying important biomarkers (12). Using these algorithms can help in the development of predictive models that have the potential to transform the way we diagnose and manage LN.

In this study, we use a combination of single-cell sequencing and machine learning technologies to comprehensively investigate the transcriptional and immune profiles of LN, identifying key immune-related genes that could serve as potential therapeutic targets. With the help of these advancing analytical techniques, our research aims to delineate the complex interactions and molecular signatures of LN, to improve outcomes for patients through more precise diagnostics and targeted therapies.

## 2 Materials and methods

### 2.1 Data processing

Single-cell RNA sequencing (scRNA-seq) data of twenty-four patients with lupus nephritis (LN) and ten control samples were obtained from ImmPort (13). Bulk RNA datasets were collected from the GEO database. Four cohorts of bulk RNA datasets of LN patients were included in this study (**Supplementary Table S1**) (14–17). Immune-related genes (IRGs) were acquired from ImmPort (18).

### 2.2 Single-cell data analysis of LN patients

Seurat was used for filtering and subsequent clustering (19). Twenty-four LN patients and ten control samples from living donor kidney biopsies were analyzed. Cells with RNA feature counts less than 500 or greater than 5000 and mitochondrial content exceeding 25% were excluded as poor-quality cells. The T-SNE algorithm was applied for visualization (20), and batch effect correction was performed using the “RunHarmony” function (21). Cell subtypes were annotated according to cell markers from the original study (13). Differential expression analysis was performed using the Wilcoxon method to identify genes with significant expression differences (DEGs) between groups, setting adjusted P values to 0.05 and the absolute log_2_FC value to >1.

### 2.3 Non-negative Matrix Factorization (NMF) and Meta-Program detection of leukocytes in LN patients

For the analysis of leukocytes in LN samples, the consensus Non-negative Matrix Factorization (cNMF) algorithm was utilized (https://github.com/dylkot/cNMF) (22). The optimal number of components (k) was determined using the diagnostic plot approach from the provided tutorial (https://github.com/dylkot/cNMF). To identify nonoverlapping gene modules, we used a gene ranking algorithm. Expression program patterns were further analyzed by employing Pearson correlations and hierarchical clustering, resulting in the Meta-Programs.

### 2.4 Establishment of predictive IRG models for LN by machine learning

To predict LN more accurately and universally, we employed a comprehensive suite of twelve different machine learning algorithms. These tools help us understand complex biological data by finding patterns that humans may not easily recognize. The algorithms we used include:

1. LASSO (Least Absolute Shrinkage and Selection Operator) - Simplifies the analysis by reducing the number of data variables.
2. Ridge Regression - Analyzes data by considering various factors simultaneously to minimize errors.
3. Elastic Net (Enet) - Combines the features of LASSO and Ridge to provide a balanced analysis of the data.
4. Stepwise Generalized Linear Models (Stepglm) - Builds a model by adding or removing potential predictors one at a time based on statistical criteria.
5. Support Vector Machines (SVM) - Finds the best boundary that separates different groups of data.
6. Generalized Linear Model Boosting (GlmBoost) - Improves prediction accuracy by combining several simpler models into a more powerful one.
7. Linear Discriminant Analysis (LDA) - Helps to find a linear combination of features that characterizes or separates two or more classes of objects or events.
8. Partial Least Squares Regression (plsRglm) - Focuses on finding the relationship between input data and the response variable by extracting relevant information.
9. Random Survival Forests (RF) - A method that uses decision trees to predict the outcome over time.
10. Gradient Boosting Machines (GBMs) - Builds one tree at a time and each new tree helps to correct errors made by previously built trees.
11. Extreme Gradient Boosting (XGBoost) - An optimized version of GBM that is faster and more efficient.
12. Naive Bayes - A simple but powerful algorithm based on probabilistic logic.

### 2.5 Model construction

We began building our models using a combination of two large datasets (GSE32591 and GSE113342) that contain extensive genetic information from LN patients. This rich data helps us to train our models effectively, aiming to predict the severity and presence of LN by analyzing patterns in the expression of immune-related genes.

### 2.6 Model validation

After constructing the models, their reliability and accuracy were thoroughly evaluated using two additional independent datasets, GSE200306 and GSE81622. These datasets were chosen to test whether our models can reliably work under different conditions and with various patient groups. We assessed the performance of each model by calculating the Area Under the Receiver Operating Characteristic Curve (AUC), which measures the ability of the model to correctly classify patients with and without LN.

### 2.7 Expression validation of hub IRGs by six cohort.

The expression levels of six hub IRGs, identified as central to the predictive models, were validated using six datasets (**Supplementary Table S2**) (14, 23–25).

### 2.8 Expression validation of hub IRGs in an in-house cohort via real-time PCR analysis

We conducted expression validation of the hub IRGs within our in-house cohorts using quantitative real-time PCR. Blood samples were obtained from both healthy controls and LN patients at Shenzhen Second People’s Hospital. Participants provided written informed consent, and the study was approved by the ethics committee (Approval No. 20220824001).

Total RNA was extracted from the blood samples using the SteadyPure Quick RNA Extraction Kit (AG21025, AG, Hunan, China). LN (n=4) and control (healthy volunteers, n=4) were analyzed. Subsequent reverse transcription of RNA into cDNA was performed using the Reverse Transcription Kit (RR036A, Takara, Japan). The real-time PCR analysis was carried out with the SYBR Green Master Mix (QPK-201, TOYOBO, Japan) on a QuantStudio™ 3 Real-Time PCR System (Thermo Fisher Scientific, USA). The specific primer sequences employed are detailed in **Supplementary Table S3**.

### 2.9 Clinical correlation

To understand the clinical significance of these hub IRGs, we analyzed their correlation with critical clinical parameters such as the glomerular filtration rate (GFR), proteinuria, serum creatinine levels, and pathological stages. This analysis aimed to elucidate the biological relevance of these genes in the context of LN pathophysiology and their potential as biomarkers for disease progression and severity

### 2.10 Protein interaction network analysis of hub IRGs

We conducted a comprehensive exploration of potential protein interactions involving six hub IRGs. Using the STRING database (https://string-db.org/), we collated and integrated information on protein-protein interactions (PPIs). The PPI network was subsequently analyzed, considering only interactions with confidence scores exceeding 0.7 to ensure significance. To further our analysis and enhance visualization, we imported the pertinent data into Cytoscape (version 3.8.2). With Cytoscape, we used the cytoHubba plugin to identify the top 10 nodes, which were ranked based on the maximum clique centrality (MCC). These top nodes represented hub genes with potential significance in the network. Additionally, we used the BinGO plugin in Cytoscape to explore the Gene Ontology (GO) functional annotations related to the identified hub genes. This comprehensive approach shed light on the protein interaction landscape associated with the selected IRGs.

### 2.11 Statistical analysis

All the statistical analyses of the single-cell and bulk-RNA data were performed with R (version 4.3.1). A P value less than 0.05 was considered to indicate statistical significance.

## 3 Results

### 3.1 Identification of immune-related genes (IRGs) in lupus nephritis (LN) by single-cell analysis

**Figure 1:**
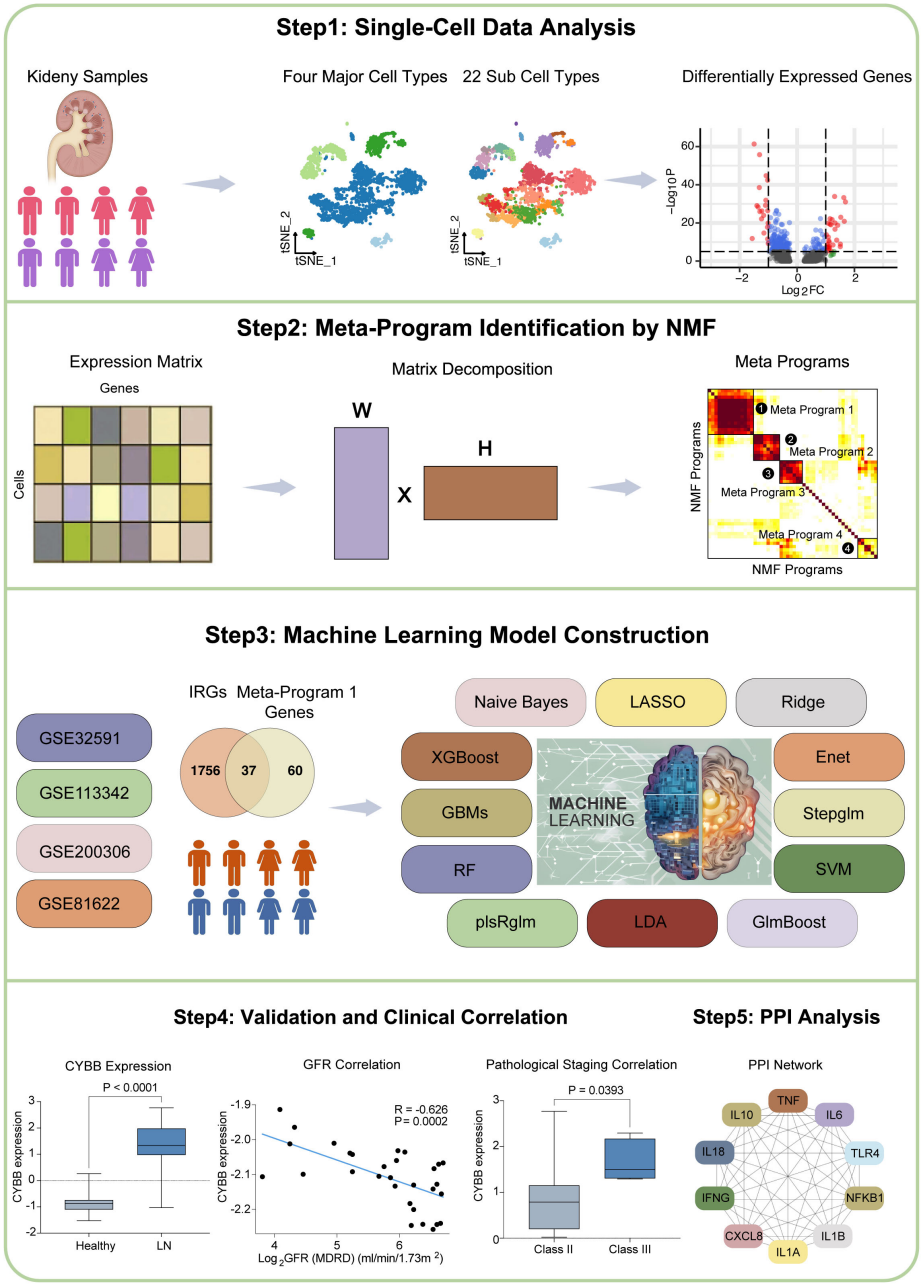
The workflow of this study.

The workflow of this study is shown in **Figure 1**. Our research can be summarized in key stages: Stage 1. Single-cell data analysis: We analyzed single-cell data from LN patients and healthy controls. Different cell types were clustered, and differential gene expression was visualized. Stage 2. Meta-Program identification: NMF revealed transcriptional programs in LN leukocytes, yielding four Meta-Programs with distinct gene sets. Stage 3. IRG Predictive Model Construction: Thirty-seven IRGs from Meta-Program 1 were used to create 102 predictive models using 12 algorithms. The top-performing models (GBM, Stepglm with Naive Bayes) included six hub IRGs. Stage 4. Validation and Clinical Correlations: Validation confirmed hub IRG upregulation in LN patients, and correlations with clinical parameters were established. Stage 5. Protein-protein interaction analysis: The interaction network of six hub IRGs was explored. Key genes were identified, and their molecular functions were characterized.

### 3.2 Analysis of single-cell dataset

In the initial phase of our investigation, we acquired a single-cell dataset including renal biopsies from 24 LN patients and 10 healthy control participants. Following quality control, normalization, and preliminary dimensionality reduction, we employed t-distributed stochastic neighbor embedding (t-SNE) algorithms to effectively distinguish cellular clusters representing the LN and control cohorts.

**Figure 2:**
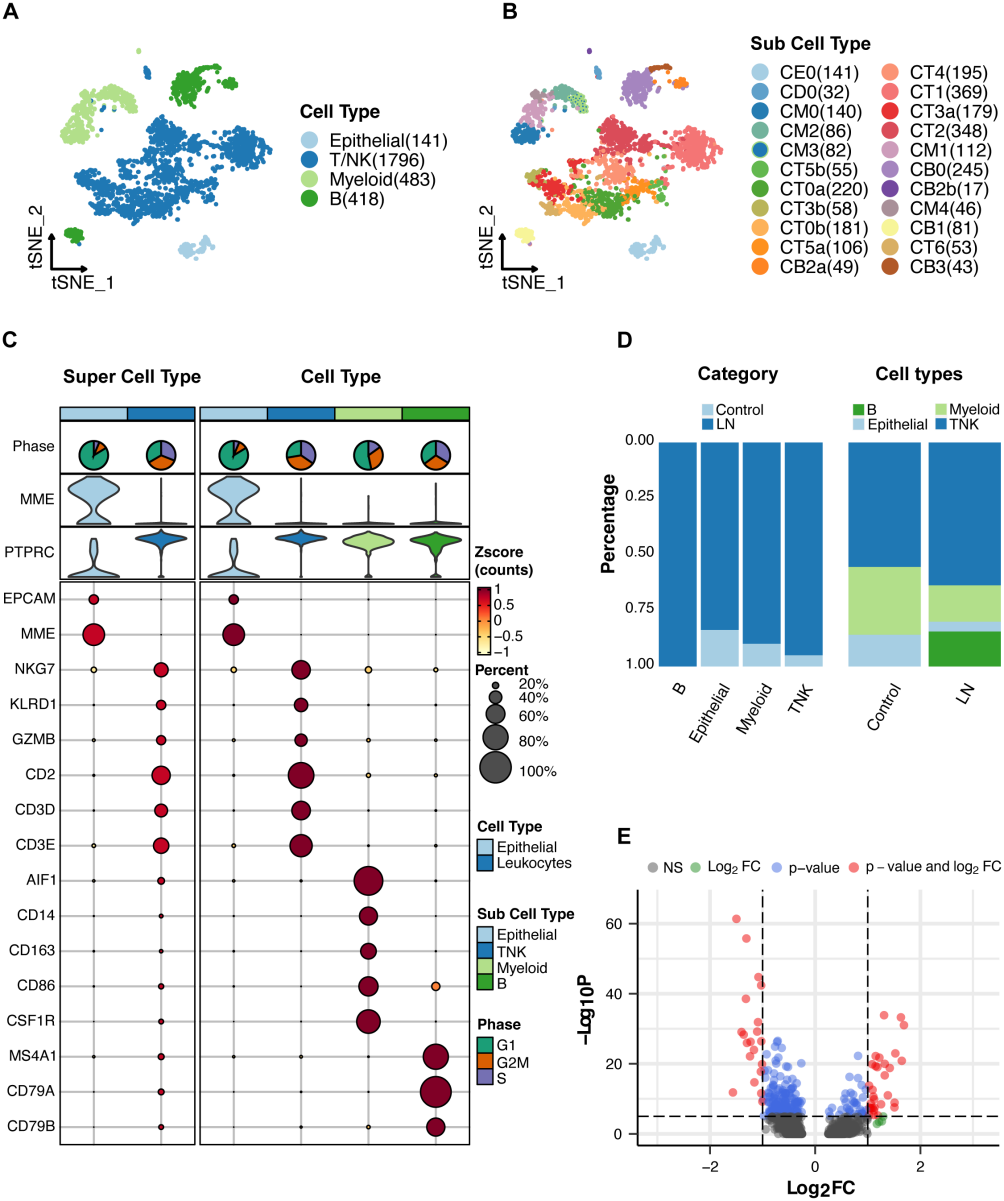
Single-cell RNA sequencing analysis of lupus nephritis (LN) tissue. **(A)** T-SNE analysis of the single-cell data showing four major cell types in the kidney samples. **(B)** T-SNE analysis of 22 subcell types. **(C)** Expression of cell markers in major cell types. **(D)** Proportion of major cell types. **(E)** A volcano plot showing the genes whose expression was upregulated or downregulated in LN patients compared with healthy participants.

Consistently, the same set of cell markers employed in the original study was retained for our analysis to categorize the four primary cell types (**Figure 2A**). These major cell types include various leukocytes, including T/NK cells, myeloid cells, and B cells, in addition to epithelial cells (**Figure 2A**). Subsequently, these major cell types were further partitioned into 22 distinct subcell types (**Figure 2B** and **Supplementary Table S4**).

For a comprehensive overview, the cell markers characterizing each cell type and their relative proportions are presented in **Figures 2C, D**. Moreover, we conducted a differential gene expression analysis, visualized in a volcano plot (**Figure 2E**), to reveal the genes exhibiting significant differences between the LN and healthy control groups. The top five upregulated genes in LN were *MX1*, *ISG15*, *IFI44L*, *EPSTI1*, and *IGHG1* (**Supplementary Table S5**). The top five downregulated genes in LN were *PRG4*, *ZBTB16*, *ALDOB*, PCK1, and *PFKFB3* (**Supplementary Table S5**). These findings collectively illuminate the cellular landscape of LN and provide crucial insights into the underlying genetic alterations. Notably, *ISG15* is a type of interferon-stimulated gene that primarily functions in the immune system. *ISG15* can also modulate immune responses by influencing the activities of immune cells.

### 3.3 Meta-Program identification of LN leukocytes

To reveal the transcriptional landscape of leukocytes in LN samples, we first eliminated epithelial cells, focusing exclusively on leukocyte-derived transcriptional signatures. Using Non-negative Matrix Factorization (NMF), we extracted and defined specific transcriptional programs unique to LN leukocytes in each sample. Our method for characterizing cell states entailed the systematic cataloging of underlying gene modules. Recent research has recognized gene modules as pivotal features defining cell states. This flexible approach accommodates the coexpression of various modules within cells, leading to the diversity of potential cell states. Our method was effective in detecting groups of genes exhibiting coexpression patterns within individual samples. To discover recurring gene modules across LN samples, we conducted comparative analyses of the gene composition within the identified modules. This approach, focusing on gene modules rather than expression matrices, helped alleviate the impact of technical variations among the samples.

**Figure 3:**
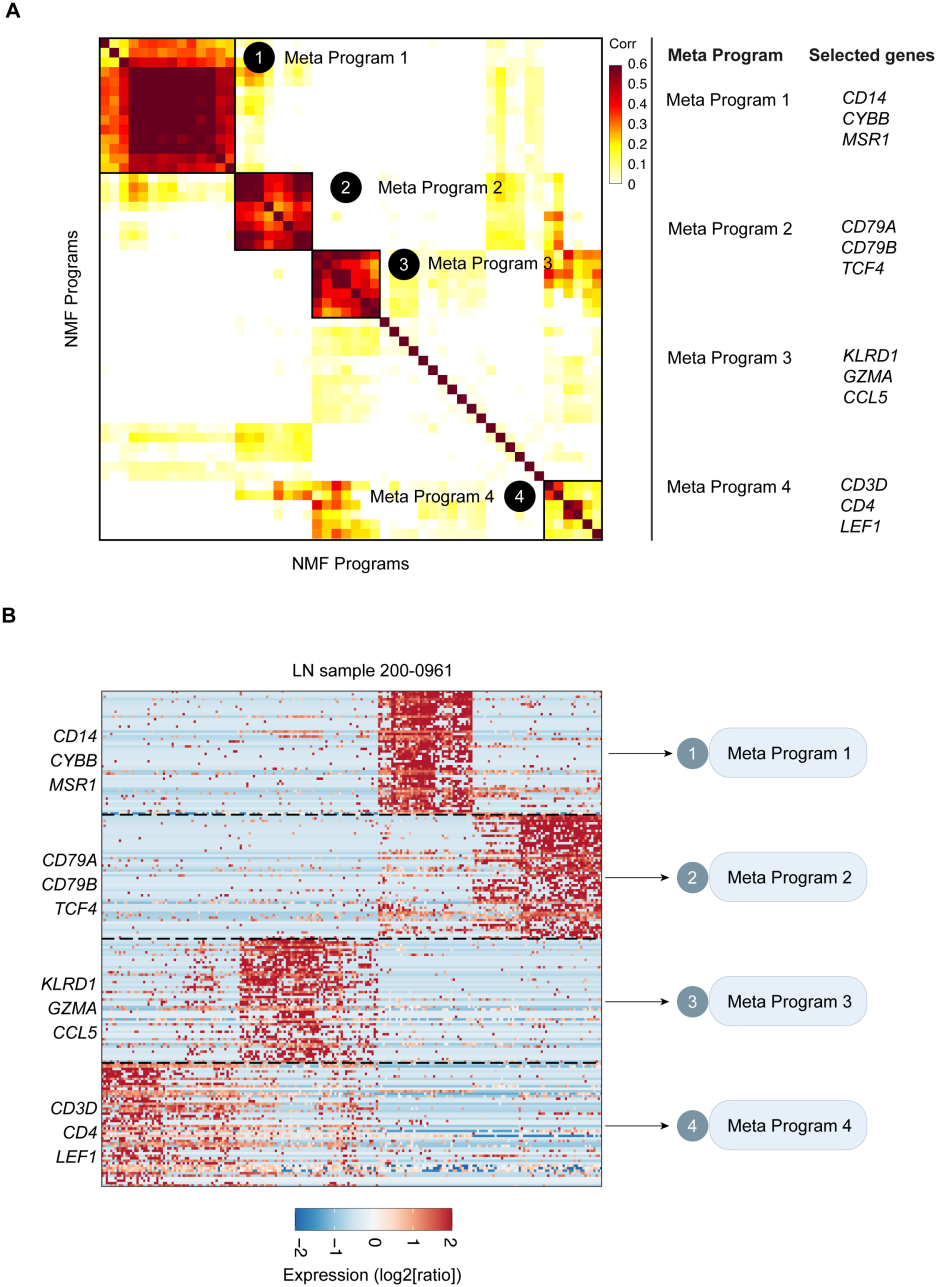
Catalog of LN gene modules in leukocytes. **(A)** Heatmap of the significance of the overlap between LN gene modules in leukocytes. Four consensus modules were identified, including Meta-Program 1, Meta-Program 2, Meta-Program 3, and Meta-Program 4. Selected genes in each Meta-Program are shown. **(B)** Heatmap of the expression levels of genes in the leukocytes of LN sample 015. Genes are ordered by their module membership (horizontal lines), and the indicated genes correspond to their consensus module annotation.

Through correlation clustering, we organized the identified expression programs into four Meta-Programs, each characterized by its top-scoring genes (**Figure 3A**). These programs were named Meta-Program 1 (comprising selected genes such as *CD14*, *CYBB*, and *MSR1*), Meta-Program 2 (encompassing selected genes such as *CD79A*, *CD79B*, and *TCF4*), Meta-Program 3 (including selected genes such as *KLRD1*, *GZMA*, and *CCL5*), and Meta-Program 4 (with selected genes such as *CD3D*, *CD4*, and *LEF1*). For example, we defined four Meta-Programs that vary among leukocytes of the patient 200–0961 (**Figure 3B**).

### 3.4 Development of the IRG predictive model of LN

**Figure 4:**
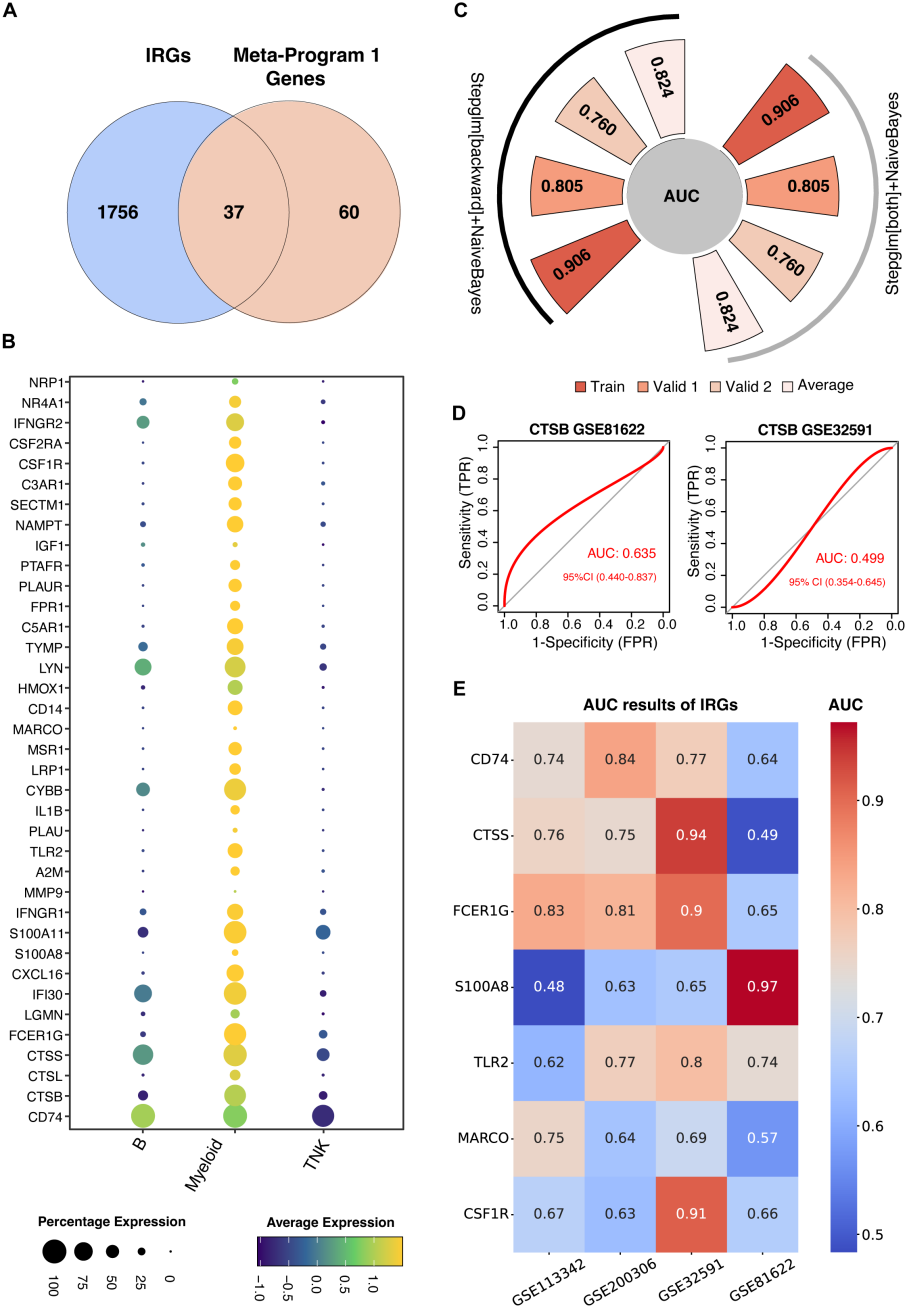
Construction of machine learning-derived prediction models. **(A)** Identification of thirty-seven genes at the intersection of Meta-Program 1 and immune-related genes (IRGs) from the ImmPort database. **(B)** Expression profiling of these 37 genes across various immune cell types. **(C)** AUC values for the best-performing predictive models using combined Stepglm [both] and Naive Bayes, and combined Stepglm [backward] and Naive Bayes algorithms. The training cohort included the datasets from GSE32591 and GSE113342; validation cohort 1 comprised GSE200306; and validation cohort 2 included GSE81622. **(D)** AUC values for *CTSB* gene in GSE32591 and GSE81622 datasets. **(E)** AUC values for IRGs expressed across all four datasets (GSE32591, GSE113342, GSE200306, GSE81622) and previously reported in studies.

To develop a predictive IRG model of LN, IRGs were identified via intersection within Meta-Program 1. As a result, 37 genes were identified (**Figure 4A**). The expression of these 37 genes in the single-cell datasets is shown in **Figure 4B**. Among the different leukocyte cell types, most of these genes were highly expressed in myeloid cells (**Figure 4B**).

We used twelve diverse machine learning algorithms, including (1) LASSO, (2) Ridge, (3) Enet, (4) Stepglm, (5) SVM, (6) GlmBoost, (7) Linear LDA, (8) plsRglm, (9) RF, (10) GBMs, (11) XGBoost, (12) Naive Bayes, to develop a robust IRG predictive model. This model demonstrated superior predictive accuracy, as evidenced by high AUC values, in both training datasets (comprising GSE32591 and GSE113342) and validation datasets (GSE200306 and GSE81622), shown in **Supplementary Figure S1**. In total, we constructed 102 machine learning-based predictive models. The top-performing models were those combining GBM and Stepwise GLM [both] with Naive Bayes, and Stepwise GLM [backward] with Naive Bayes.

The GBM model utilized fifteen genes (*CYBB, IFNGR1, MSR1, CSF1R, CTSS, PLAUR, CD14, IL1B, PTAFR, S100A8, FCER1G, CD74, TLR2, MARCO*, and *PLAU*) highlighting their pivotal role in LN diagnostics (**Supplementary Figure S1**). Conversely, a more streamlined model involving six genes (*CD14, CYBB, IFNGR1, IL1B, MSR1*, and *PLAUR*) was used for the combined Stepwise GLM and Naive Bayes approach. This refined model achieved AUCs of 0.906 and 0.805 in the training and validation cohorts, respectively, reinforcing their diagnostic efficacy (**Figure 4C**). These optimized models focus on six hub genes, offering a more practical approach for clinical application due to their simplified yet effective feature set.

Further validation using blood samples retained significant diagnostic power, with an AUC of 0.760, emphasizing the potential for a noninvasive diagnostic methodology suitable for early detection and continuous monitoring of LN (**Figure 4C**).

To test the performance of our predictive model, we evaluated the AUC of previously identified IRGs (**Table 1**), such as *CTSB*, which showed lower predictive values in comparison (**Figure 4D**). Other previously identified IRGs including *CD74, CTSS, FCER1G, S100A8, TLR2, MARCO, CSF1R* also showed lower predictive values compared with our model (**Figure 4E**). This comparative analysis confirms the superior predictive capability of our model over previously reported IRGs.

**Table 1:** Description of IRGs used to construct the prediction model in this study and reported by previous studies.

No	Gene	Full Name	Reference
IRGs used to construct the prediction model in this study			
1	CD14	CD14 Molecule	(26–29)
2	CYBB	Cytochrome B-245 Beta Chain	(30)
3	IFNGR1	Interferon Gamma Receptor 1	(31)
4	IL1B	Interleukin 1 Beta	(6, 32)
5	MSR1	Macrophage Scavenger Receptor 1	(33)
6	PLAUR	Plasminogen Activator, Urokinase Receptor	NA
Other IRGs reported by previous studies			
1	CTSB	Cement Treated Sub-Base	(28)
2	CD74	Cluster of Differentiation 74	(34)
3	CSF1R	Colony Stimulating Factor 1 Receptor	(35, 36)
4	CTSS	Cathepsin S	(37)
5	FCER1G	Fc Epsilon Receptor Ig	(38, 39)
6	MARCO	Macrophage Receptor With Collagenous Structure	(38)
7	S100A8	S100 Calcium Binding Protein A8	(40–43)
8	TLR2	Toll Like Receptor 2	(44–46)

**Figure 5:**
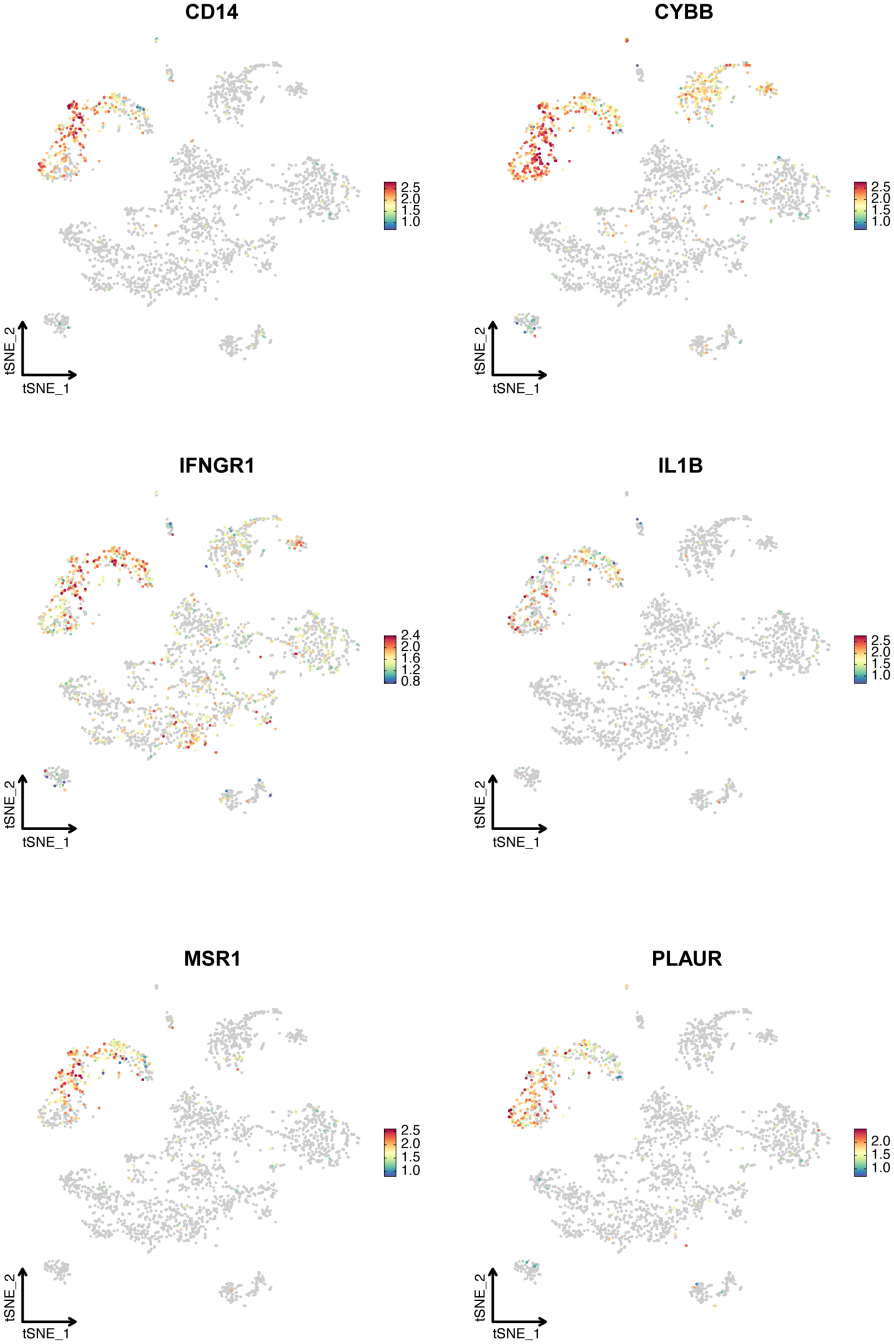
The expression of six hub IRGs identified by Stepglm plus Naive Bayes algorithm in the single-cell dataset. T-SNE plot showed the expression of *CD14, CYBB, IFNGR1, IL1B, MSR1*, and *PLAUR*.

**Figure 5** displays the expression of six hub IRGs across single-cell datasets, with notable expression primarily in myeloid cells and varying expression in B and T/NK cells, illustrating their significant role in LN.

### 3.5 Validation and clinical correlation of the six hub IRGs

**Figure 6:**
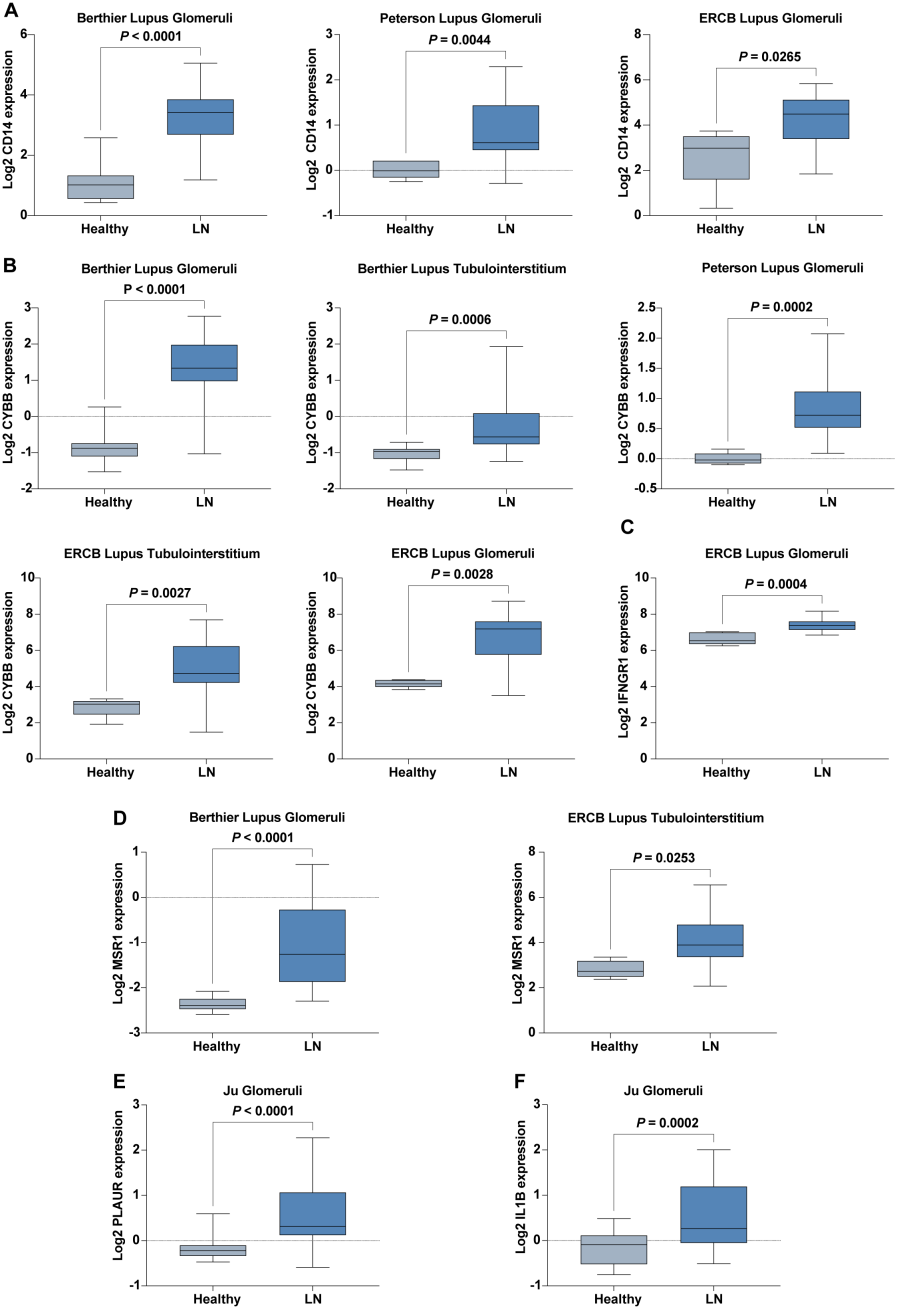
Expression of Six Hub IRGs in Six Validation Cohorts. **(A)** Expression of *CD14* across Berthier Lupus Glomeruli, Peterson Lupus Glomeruli, and ERCB Lupus Glomeruli cohorts. **(B)** Expression of *CYBB* across Berthier Lupus Glomeruli, Berthier Lupus Tubulointerstitium, Peterson Lupus Glomeruli, ERCB Lupus Tubulointerstitium, and ERCB Lupus Glomeruli cohorts. **(C)** Expression of *IFNGR1* in the ERCB Lupus Glomeruli cohort. **(D)** Expression of *MSR1* in Berthier Lupus Glomeruli and ERCB Lupus Tubulointerstitium cohorts. **(E)** Expression of *PLAUR* in the Ju Glomeruli cohort. **(F)** Expression of *IL1B* in the Ju Glomeruli cohort.

We conducted extensive validations using six cohorts and an additional in-house cohort to confirm the expression patterns of six hub IRGs: *CD14, CYBB, IFNGR1, IL1B, MSR1*, and *PLAUR*. These IRGs were found to be significantly upregulated in LN patients compared to healthy controls, underscoring their vital roles in the pathology of LN and affirming their robustness across diverse populations (**Figures 6A–F**).

**Figure 7:**
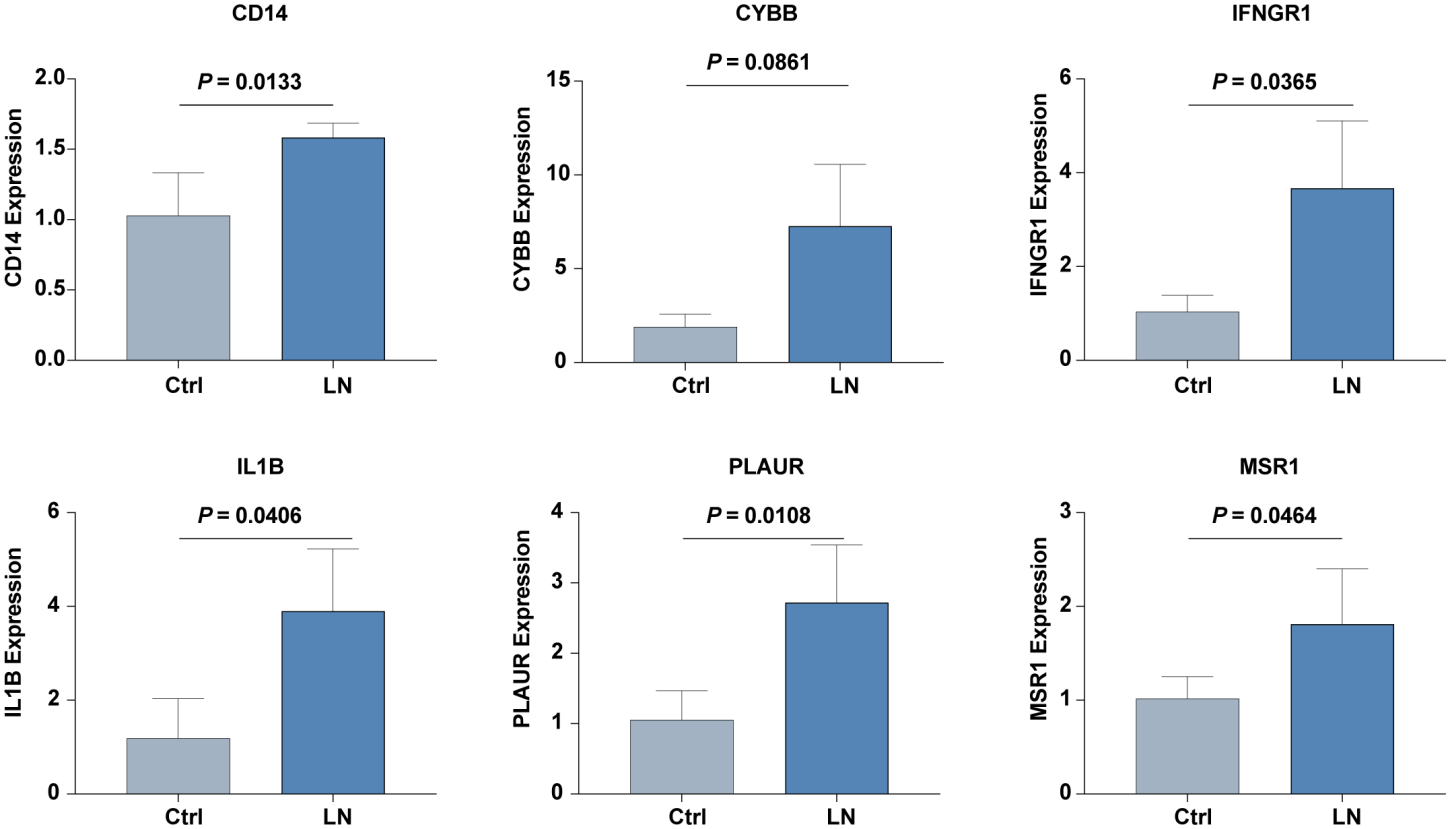
The mRNA expression level of six hub IRGs (*CD14, CYBB, IFNGR1, IL1B, PLAUR*, and *MSR1*) in our inhouse cohorts of LN and control was examined by real-time PCR analysis.

Further validation in our in-house cohort proved these findings, demonstrating consistent upregulation of six hub IRGs: *CD14, CYBB, IFNGR1, IL1B, PLAUR*, and *MSR1*. This consistency across different cohorts highlights the reliability of these IRGs as biomarkers for LN, reinforcing their potential utility in clinical diagnostics and therapeutics (**Figure 7**).

These results not only validate the significant upregulation of these hub genes in patients with LN but also emphasize the consistency of these expression patterns across varied cohort settings, enhancing the credibility and applicability of these IRGs in the broader context of LN research and patient care.

**Figure 8:**
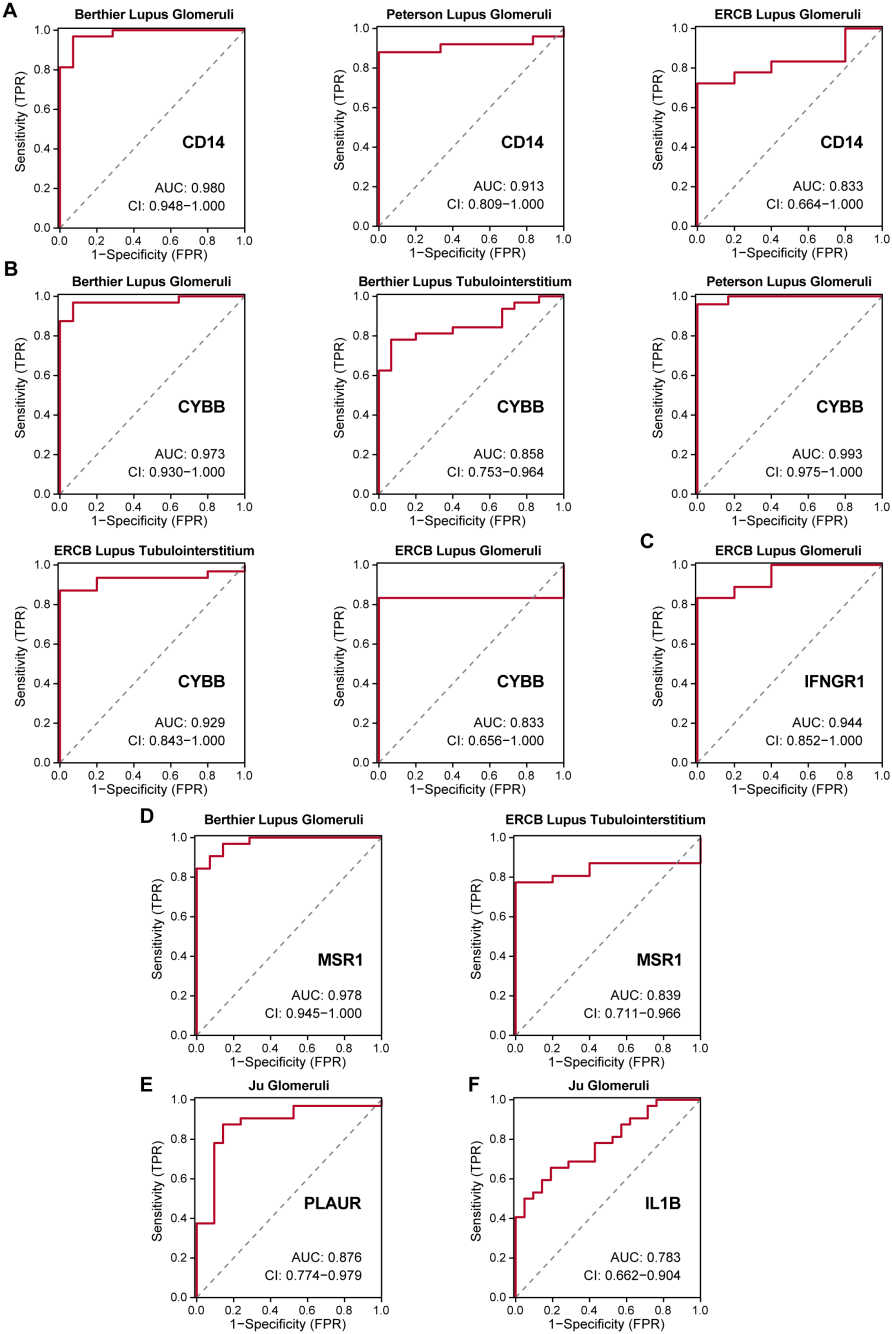
ROC curves of six hub IRGs in six validation cohorts. **(A–E)** Strong diagnostic values were demonstrated for LN with AUC>0.8 for *CD14, CYBB, IFNGR1, MSR1*, and *PLAUR*. **(F)** Moderate diagnostic value for *IL1B* with an AUC of 0.783.

ROC curve analysis further demonstrated the strong diagnostic potential of these IRGs, with most exhibiting AUC values greater than 0.8, confirming their utility in accurately diagnosing LN across diverse patient cohorts (**Figures 8A–E**). Only *IL1B* showed a moderate diagnostic value (AUC = 0.783, **Figure 8F**).

**Figure 9:**
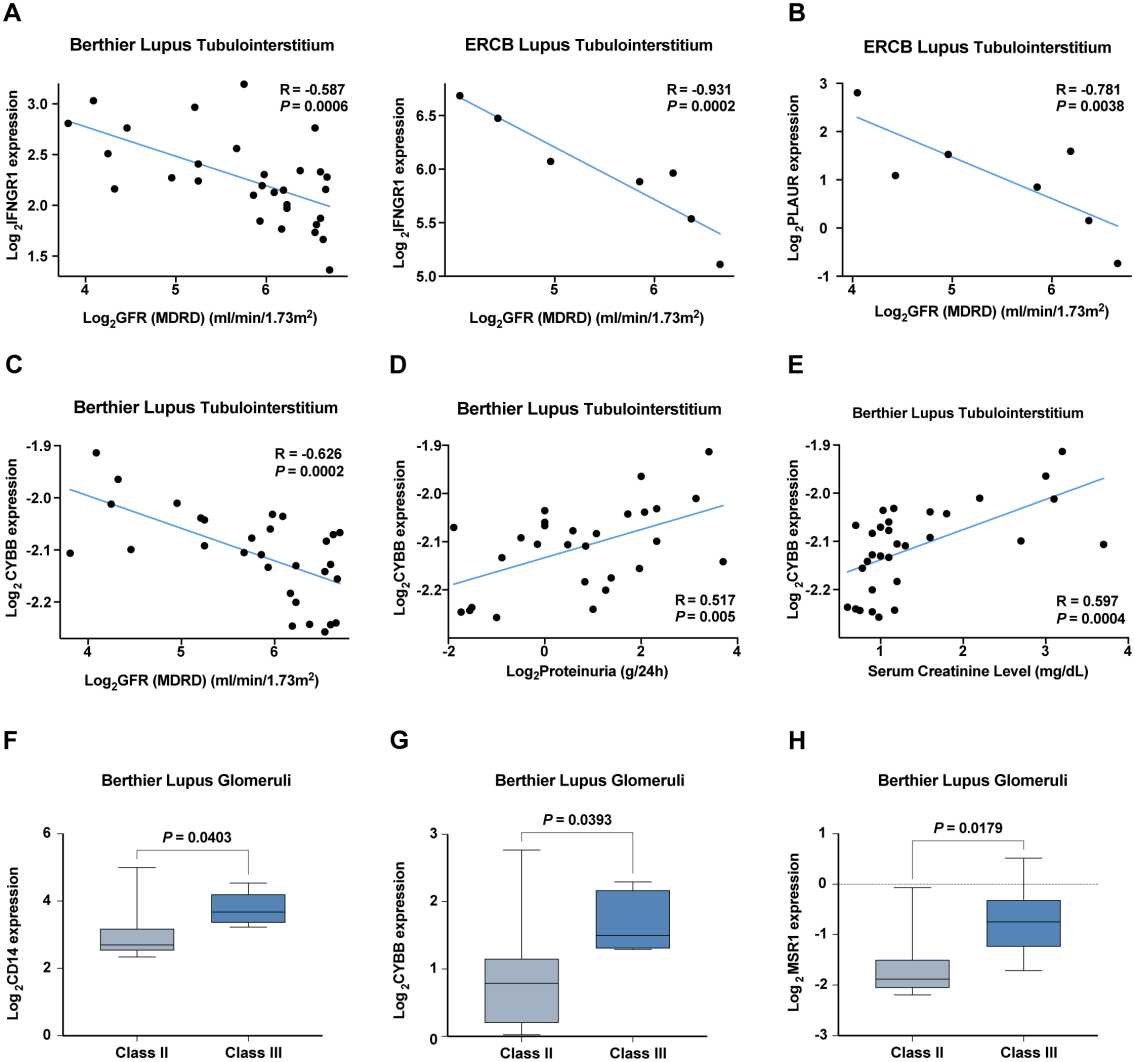
Correlation analysis of hub IRGs with renal function and pathological staging in six validation cohorts. **(A–C)** Negative correlation of **(A)** *IFNGR1*, **(B)** *PLAUR*, and **(C)** *CYBB* with the glomerular filtration rate (GFR). **(D, E)** Positive correlation of *CYBB* with **(D)** proteinuria and **(E)** serum creatinine levels. **(F–H)** Increased expression of **(F)** *CD14*, **(G)** *CYBB*, and **(H)** *MSR1* in pathological stage Class III compared with Class II.

We also explored the clinical relevance of these IRGs by analyzing their correlations with key renal function indicators such as the glomerular filtration rate (GFR), proteinuria, and serum creatinine levels across patient cohorts. Notably, expressions of *IFNGR1, PLAUR*, and *CYBB* negatively correlated with GFR, while *CYBB* also showed a positive correlation with proteinuria and serum creatinine levels, indicating their potential involvement in LN pathophysiology (**Figures 9A–E**). Increased expression of CD14, CYBB, and MSR1 was identified in pathological stage Class III compared with Class II (**Figures 9F–H**).

### 3.6 Protein-protein interaction analysis of the six hub IRGs

Next, we analyzed the intricate landscape of potential protein interactions among the six hub IRGs that we identified. Using the STRING database (https://string-db.org), we curated and amalgamated the protein-protein interaction (PPI) data. The resulting PPI network was analyzed, with only interactions with confidence scores exceeding 0.7 considered to ensure their biological relevance.

**Figure 10:**
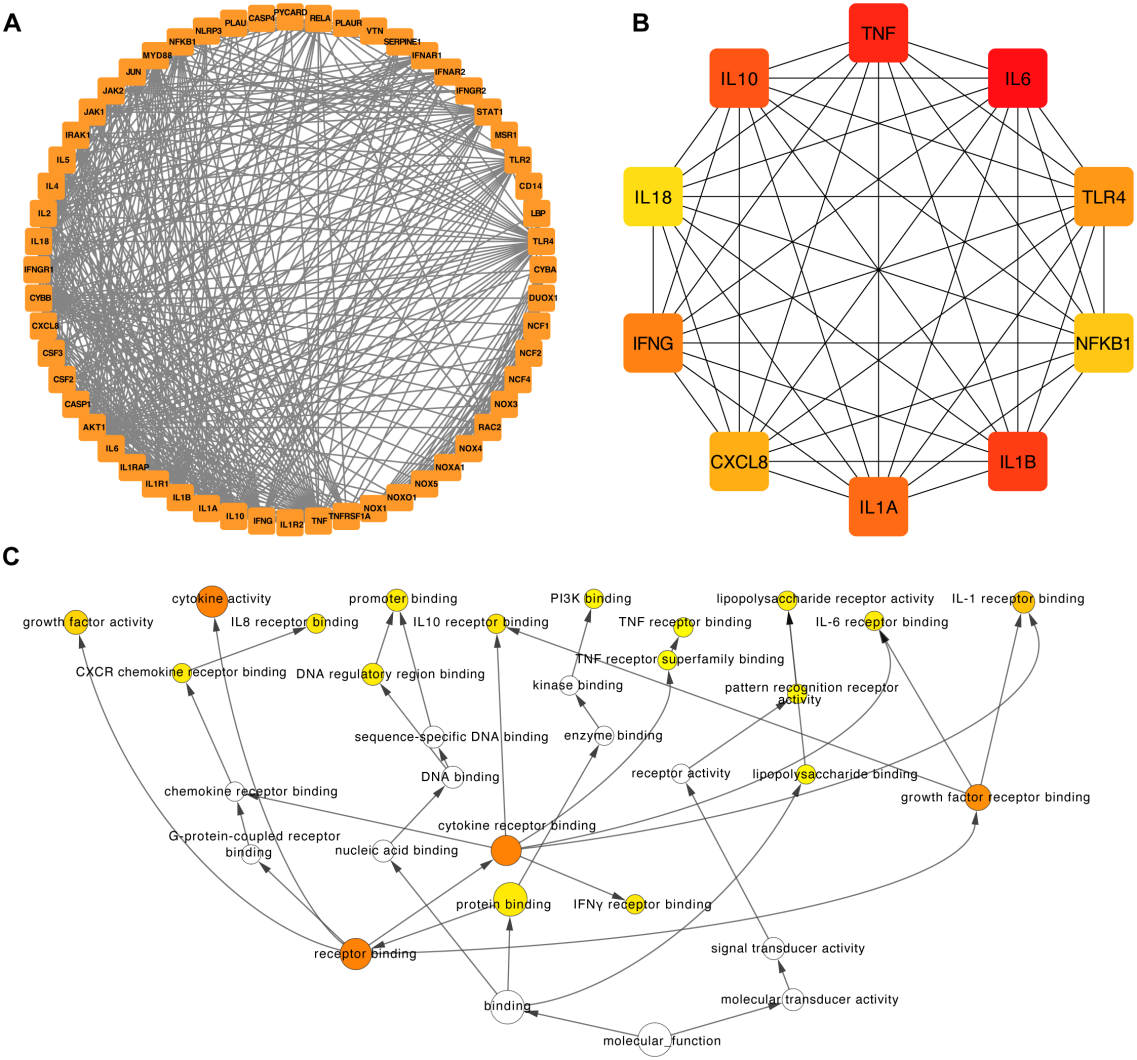
Construction of the PPI network and functional enrichment analysis of the six hub IRGs. **(A)** The PPI network was constructed based on 50 genes closely related to the six hub IRGs. **(B)** The top ten hub genes of the PPI network. **(C)** Functional enrichment of the top ten hub genes of the PPI network by BINGO.

To improve our understanding of the network and facilitate interpretation, we imported the relevant data into Cytoscape (**Figure 10A**). Within Cytoscape, we used the cytoHubba plugin to pinpoint the top 10 nodes, which were ranked based on their maximum clique centrality (MCC). These top nodes were subsequently identified as 10 hub genes (including *IL6*, *TNF*, *IL1B*, *IL10*, *IL1A*, *IFNG*, *TLR4*, *CXCL8*, *NFKB1*, and *IL18*), indicating the potential importance of the interleukin family and chemokines within the interaction network (**Figure 10B**).

Furthermore, we used the capabilities of the BinGO plugins within Cytoscape to unravel the Gene Ontology (GO) functional annotations associated with the 10 hub genes (**Figure 10C**). The results showed that the molecular functions of these genes were mainly associated with receptor binding, protein binding, interferon-gamma receptor binding, and growth factor receptor binding (**Figure 10C**).

## 4 Discussion

Lupus nephritis (LN) represents a complex and challenging aspect of systemic lupus erythematosus (SLE) and is associated with high morbidity and mortality rates (2). Current treatment strategies predominantly rely on immunosuppressive regimens, which often have adverse effects and may lead to treatment resistance. The primary objective of this study was to improve our understanding of LN and identify potential biomarkers and therapeutic targets to improve patient outcomes.

The advent of single-cell sequencing technology has revolutionized our capacity to explore the intricate biological underpinnings of LN (47). In addition to using dimensionality reduction techniques for cellular clusters, we carried out a more profound exploration of the transcriptional landscape of LN leukocytes. Building upon this foundation, our study utilized Non-negative Matrix Factorization (NMF) to understand the transcriptional landscape of LN leukocytes, focusing exclusively on leukocyte-derived transcriptional signatures after eliminating epithelial cell data. This methodological approach facilitated the identification of specific transcriptional programs unique to LN leukocytes, leading to the systematic cataloging of underlying gene modules. Our analysis successfully organized these expression programs into four Meta-Programs, each characterized by its top-scoring genes such as *CD14, CYBB*, and *MSR1* in Meta-Program 1, enriching our understanding of their roles in LN pathogenesis. Particularly noteworthy was the recognition of certain immune-related genes (IRGs) as crucial genes within these Meta-Programs.

A previous study highlighted the local activation of B cells in LN kidneys, which correlated with an age-associated B-cell signature (48). This observation aligns with our work in which we identified a cluster of B-cell-related genes (*CD79A* and *CD79B*) within Meta-Program 2, emphasizing the potential involvement of B cells in LN pathogenesis. The detection of a prominent interferon response across immune cell subsets in their study aligns with our emphasis on the impact of interferon signaling in LN. Our results also indicate an intricate relationship between interferon-responsive genes (such as *ISG15*) and LN pathogenesis, further supporting the importance of targeting this pathway for therapeutic interventions.

The identification of *CXCR4* and *CX3CR1* in previous studies highlights the crucial roles of these chemokine receptors in the context of LN (13). Our protein-protein interaction (PPI) analysis revealed interactions between our identified hub IRGs and other immune genes, which raises the possibility of a connection between these IRGs, *CXCL8*, and the receptors *CXCR4* and *CX3CR1*. This interconnectedness could indicate a complex signaling network within LN pathogenesis, where *CXCL8*, *CXCR4*, and *CX3CR1* may interact to regulate immune responses and cellular dynamics in the kidney.

*CXCL8*, known for its role in inflammatory responses and chemotaxis, may contribute to the recruitment of immune cells to the kidney, potentially via interactions with *CXCR4* and *CX3CR1*. The specific roles and implications of these interactions warrant further investigation to elucidate how they collectively impact the development and progression of LN. Elucidating these connections could uncover novel therapeutic targets and deepen our understanding of the intricate mechanisms underlying this complex autoimmune disease.

Our study uses advanced single-cell RNA sequencing and machine learning technologies to deepen our understanding of LN. Through comprehensive analysis, we have developed robust predictive models that significantly advance the diagnostic capabilities for LN. We successfully identified 37 IRGs that were highly expressed in myeloid cells among other leukocyte types. Utilizing a diverse array of twelve machine learning algorithms, we constructed 102 predictive models. The high-performance models, particularly those integrating Generalized Boosted Regression Models (GBM) with Stepwise GLM and Naive Bayes, highlighted the critical role of a concise set of IRGs in LN diagnostics. These models demonstrated superior predictive accuracy, as evidenced by AUC values exceeding 0.8 in both training and validation cohorts. This analysis confirms the enhanced capability of our approach over traditional methods and previously identified IRGs, as detailed comparisons show lower predictive values for other known IRGs.

The streamlined models that focus on six hub IRGs (*CD14, CYBB, IFNGR1, IL1B, MSR1*, and *PLAUR*) emphasize their pivotal roles and potential as biomarkers for noninvasive diagnostics, making them particularly useful for clinical applications. The success of these models in blood sample validations, achieving an AUC of 0.760, underscores the feasibility of using these biomarkers for early detection and continuous monitoring of LN.

The validation of these six hub IRGs in six diverse patient cohorts further solidified their diagnostic value, as most exhibited strong diagnostic potential with AUC values greater than 0.8. This broad validation supports the robustness of our findings and their applicability across different clinical settings. Additionally, the correlation analysis with key renal function indicators, reveals the clinical relevance of these IRGs, particularly their associations with glomerular filtration rate (GFR), proteinuria, serum creatinine levels, and pathological stages indicating their direct involvement in the pathophysiology of LN.

This research not only identifies critical biomarkers for LN but also sets a foundation for future studies to explore accessible, patient-friendly diagnostic methods. The identification of these hub IRGs and their incorporation into predictive models offer promising avenues for timely interventions and improved patient outcomes, aligning with the goals of precision medicine.

Intriguingly, the six hub IRGs we identified, namely, *CD14*, *CYBB*, *IFNGR1*, *IL1B*, *MSR1*, and *PLAUR*, have been the subject of previous research in various contexts. *CD14*, for instance, plays a role in immune regulation and is associated with innate immune responses. It has also been previously linked to systemic autoimmune diseases, such as LN. The cytochrome B-245 beta chain (*CYBB*) encodes a key component of the NADPH oxidase complex and contributes to reactive oxygen species (ROS) production, which is implicated in the pathogenesis of LN (30). *IFNGR1*, a crucial component of the interferon-gamma signaling pathway, is known to be associated with SLE (49). *IL1B* is a proinflammatory cytokine that has been widely studied in the context of LN and contributes to the inflammatory processes that characterize this disease (6). *MSR1*, a scavenger receptor, is expressed by macrophages and is implicated in immune responses and autoimmunity (33). *PLAUR*, a receptor for urokinase-type plasminogen activator (uPA), has been investigated in the context of autoimmune disorders and tissue remodeling (50).

Moreover, we employed the BinGO plugin in Cytoscape to delve deeper into the functional relevance of the identified hub genes. By revealing the Gene Ontology (GO) functional annotations associated with these hub genes, we obtained valuable insights into their potential molecular functions. Our analysis revealed that the 10 hub genes (including *IL6*, *TNF*, *IL1B*, *IL10*, *IL1A*, *IFNG*, *TLR4*, *CXCL8*, *NFKB1*, and *IL18*) we identified are primarily associated with several key molecular functions. These functions included receptor binding, protein binding, interferon-gamma receptor binding, and growth factor receptor binding. These findings provide a comprehensive understanding of the molecular roles these genes play in the context of LN. Receptor binding and protein binding functions are indicative of the role of the hub genes in interactions with other molecules, signaling pathways, and cellular processes. The presence of interferon-gamma receptor binding and growth factor receptor binding functions among the hub genes highlights their involvement in immune responses and signaling cascades, which are pivotal in LN pathogenesis. These results not only enhance our understanding of the hub genes involved in LN but also suggest potential therapeutic targets. Targeting these specific molecular functions could provide innovative approaches for the development of novel treatments and interventions for LN, ultimately improving patient outcomes and prognosis.

Our protein-protein interaction analysis, utilizing the STRING database and visualized in Cytoscape, has expanded our understanding of the molecular interactions at play in LN. By examining the interactions among six hub IRGs, we have elucidated a complex network of protein interactions that underscores the interconnected nature of immune responses in LN. This analysis, depicted in **Figure 9A**, used the cytoHubba plugin to highlight the top ten hub genes including *IL6, TNF, IL1B, IL10, IL1A, IFNG, TLR4, CXCL8, NFKB1*, and *IL18*. These genes are predominantly associated with the interleukin family and chemokines, suggesting their central role in mediating inflammatory responses in LN.

The functional annotations derived from the BinGO plugin in Cytoscape, as shown in **Figure 9C**, provided deeper insights into the roles these hub genes play in disease pathology. These genes are primarily involved in critical molecular functions such as receptor binding, protein binding, interferon-gamma receptor binding, and growth factor receptor binding. Such functions are crucial for the modulation of immune responses and signal transduction pathways that are pivotal in the development and progression of LN.

The detailed analysis of these gene functions highlights how these hub genes interact with other molecules and participate in complex signaling cascades that drive the pathophysiology of LN. For instance, the receptor and protein binding activities of these genes suggest their involvement in cell-cell interactions and immune regulation, which are essential for the coordination of an effective immune response. Moreover, the specific roles of interferon-gamma receptor binding and growth factor receptor binding underscore the significance of these genes in immune modulation and tissue repair processes, respectively.

Our study has made significant advancements in understanding LN through the identification of hub IRGs and the development of predictive models. However, several limitations must be carefully considered to fully appreciate the scope and application of our findings. (1) Variable Quality of Single-Cell Sequencing Data: Single-cell RNA sequencing is a powerful tool for dissecting cellular heterogeneity in diseases like LN. However, its susceptibility to data quality variability can impact the accuracy of gene expression analysis and the subsequent predictive modeling. We have implemented stringent quality controls and normalization procedures to mitigate these effects, yet the inherent limitations in data consistency remain a challenge for the reproducibility and reliability of our results. (2) Sensitivity of Machine Learning Algorithms: The algorithms used in our predictive models are sensitive to the characteristics of the training datasets. This sensitivity can affect both the accuracy and the generalizability of our findings, necessitating cautious interpretation and application in diverse clinical settings. Our study’s predictive models were validated with carefully selected cohorts known for their data quality and relevance to LN. However, these cohorts may not fully represent the global LN patient population, potentially limiting the generalizability of our findings. (3) Need for Extensive Validation: The clinical translation of our identified biomarkers and predictive models requires rigorous validation across independent cohorts and varied populations to confirm their effectiveness and reliability in clinical diagnostics. This process is crucial to reduce the risk of misdiagnosis or inappropriate treatment and to ensure that our models perform reliably in real-world settings. (4) Clinical Applicability and Longitudinal Validity: Although our models show promise, their practical implementation is non-trivial and demands further extensive clinical validation. Additionally, while we have identified promising correlations between certain hub genes and clinical outcomes, these findings are primarily based on cross-sectional data. Longitudinal studies are necessary to establish causality and determine the long-term reliability of these biomarkers in predicting disease progression. (5) Understanding Protein-Protein Interactions: Our protein-protein interaction analyses have provided valuable insights into the potential mechanisms linking identified IRGs with LN. However, a deeper understanding of the functional implications of these interactions is needed. This requires further experimental validation of predicted interactions and their roles in LN pathophysiology, which could inform potential therapeutic targets. (6) Potential for Enhancing Early Detection and Personalized Medicine: Despite these challenges, the biomarkers identified hold significant potential for enhancing early detection and personalizing disease management. Their integration into clinical practice must be approached with a deep understanding of the biological complexity of LN and a commitment to continued research to elucidate the underlying mechanisms that govern their roles in disease pathophysiology.

In conclusion, our study provides a valuable foundation for a deeper understanding of the genetic basis of LN. By using complex transcriptional programs, machine learning-based predictive models, and protein-protein interactions within the LN, we aspire to help clinicians and researchers make well-informed decisions and formulate more effective treatments. These findings underscore the clinical potential of these hub IRGs in assessing disease progression and guiding personalized treatment decisions.

## Data Availability

The datasets presented in this study can be found in online repositories. The names of the repository/repositories and accession number(s) can be found in the article/**Supplementary Material**.

## Supplementary material

Supplementary Figure 1 The AUC of 102 machine learning algorithm combinations in the training and validation cohorts. The training cohort included the combination of the GSE32591 and GSE113342 datasets. The validation cohort included GSE200306 and GSE81622 datasets.
