# Supplementary figures and images for: Applying 12 machine learning algorithms and Non-negative Matrix Factorization for robust prediction of lupus nephritis
Source: Front Immunol. 2024 Aug 19;15:1391218. doi: 10.3389/fimmu.2024.1391218 (PMC11366613; doi:10.3389/fimmu.2024.1391218)

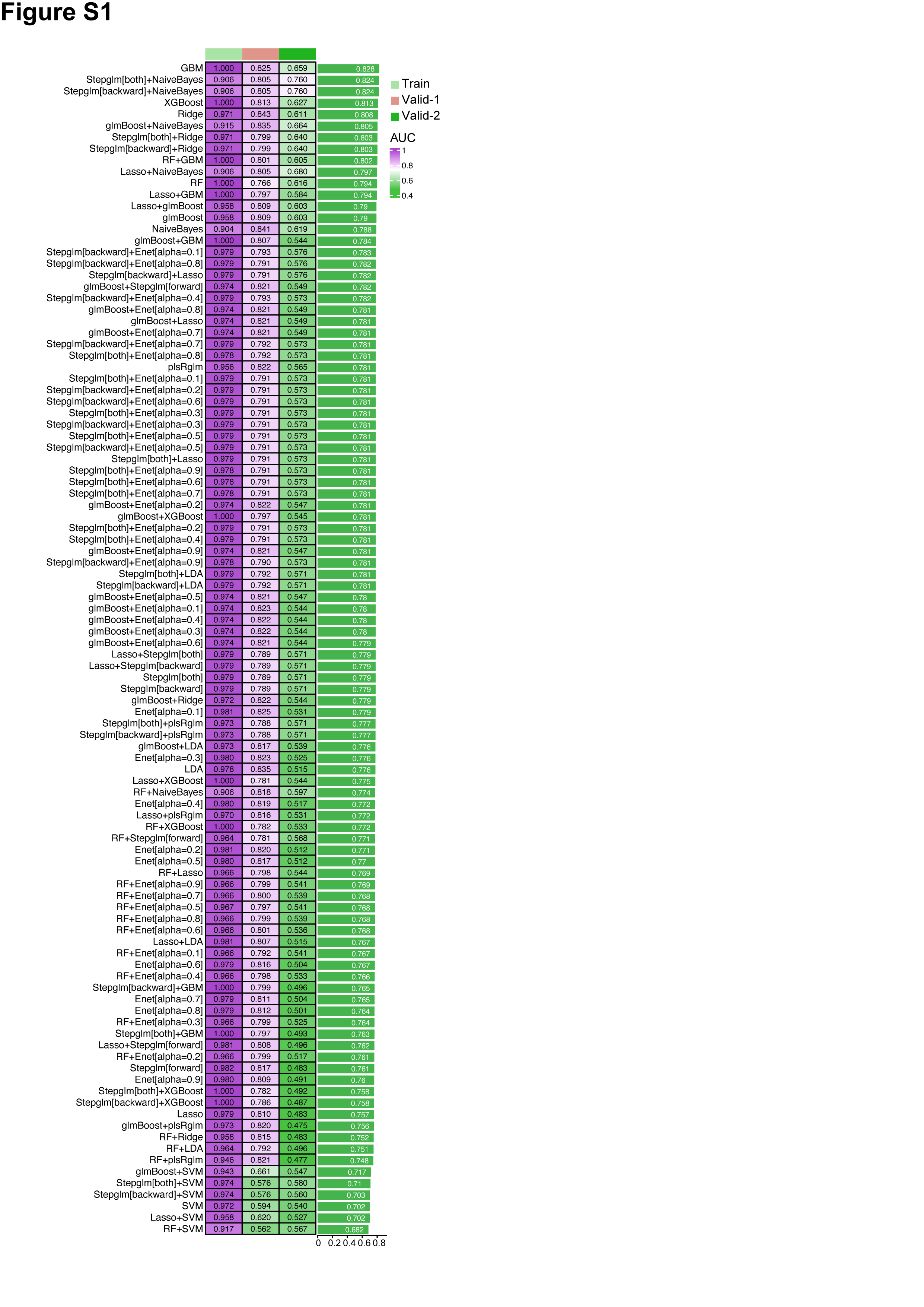

Supplement: Supplementary Figure 1 — The AUC of 102 machine learning algorithm combinations in the training and validation cohorts. The training cohort included the combination of the GSE32591 and GSE113342 datasets. The validation cohort included GSE200306 and GSE81622 datasets. [file Image1.tif]
